# Genomic Data and Disease Forecasting: Application to Type 2 Diabetes (T2D)

**DOI:** 10.1371/journal.pone.0085684

**Published:** 2014-01-17

**Authors:** Lawrence Sirovich

**Affiliations:** Center for Studies in Physics & Biology, Rockefeller University, New York, New York, United States of America; Thomas J. Watson Research Center, United States of America

## Abstract

A general approach is presented for the extraction of a classifier of disease risk that is latent in large scale disease/control databases. Novel features are the following: (1) a data reorganization into a regularized *standard* form that emphasizes individual alleles instead of the single nucleotide polymorphism (Snp) allele pair to which they belong; (2) from this a procedure that significantly enhances the discovery of high value genomic loci; (3) an investigative analysis based on the hypothesis that disease represents a very small signal (small signal-to-noise) that is latent in the data. The resulting analyses applied to the FUSION T2D database leads to the polling of thousands of genomic loci to classify disease. This large genomic kernel of loci is shared by non-diabetics at nearly the same high level; but a small well defined separation exists and it is speculated that this might be due to unconventional disease mechanisms. Another analysis demonstrates that the FUSION database size limits its disease predictability, and only one third of the resulting classifier loci are estimated to relate to T2D. The remainder is associated with hidden features that might contrast the disease and control populations and that more data would eliminate.

## Introduction

In rough approximation about 99.5% of the 3 billion DNA base pairs of the human genome are shared by all homosapiens. Somatic cells contain two copies, paternal and maternal, and so about 99.5% of the pairs are homozygous. The remaining pairs appear as two alleles and are termed single nucleotide polymorphisms (Snps): based on the criterion that the rarer of the two alleles is present in greater than 1% of the *population* [1]. This heterozygous content of the Snps is generally regarded as the determinant of human diversity including the potential for acquiring diseases [2].

Genome wide association studies (GWAS) refer to extensive investigations, past and present, that endeavor to find correlations between clinically diagnosed diseases (phenotype) with their Snp counterparts (genotype), and have as a goal the determination of genomic classifiers of disease risk. Location of the two Snps associated with age related macular degeneration [3] represents a triumph of this approach. Although other disease associations have been found [4] disappointment has been expressed on the paucity of DNA linkages that have been found for human diseases, particularly in the case of complex disorders [5]. The “predictive power of DNA” has also been questioned [6] (also see [7]). Additionally doubt has been raised in regard to the relationship of identified Snps with actual disease mechanisms [8].

The methods presented here for associating Snp loci with disease lie outside traditional statistical approaches [9]–[11]. Instead the present framework originates in methods that have their origin in extracting extremely weak signals (small “signal-to-noise” ratio) such as appear in optical imaging [12]–[14]. The view of the present study is that GWAS data show a weak disease signal. In a genomic setting similar methods have been applied to taxonomic studies [15], [16]. For *complex* diseases such as diabetes it may be an error to focus on the role of individual genomic loci. Instead the disease/control contrast appears to be better sought in a large genomic framework of risk loci common to the disease cohort.

A principal goal of this effort will be to isolate out of the vast collection of genomic loci, potentially in the millions, a smaller set of loci along with an appropriate nucleotide symbol at each locus which is associated with the disease. If the number of loci (and symbols) is 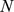 then the 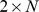 matrix of loci over genomic symbols will be referred to as the *indicator (vector)*, and the properly ordered symbols which form a word in the general sense will be termed the disease *classifier*, in the 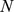 loci subspace.

The next section presents a verbal description of the Methods (technical summaries appear in the Appendix) and also provides a graphical illustration of the superior informational content of the present data reorganization; and a second illustration convincingly shows the structural difference of case/control sets in the classifier subspace. After a section describing the FUSION data, and more preliminary results, a section entitled Validation and Prediction follows. This deals with the self-consistency of the classifier that was determined in the previous section, but also points out limitations on the predictability of the classifier when applied to other data. The final Discussion section presents additional results, implications and speculations.

## Methods

### The Standard Organization

A typical database is composed of disease and control genomic records. Each such record is a sequence of symbols at Snp locations common to the database. Information on the chromosome number, chromosome location, and Snp alleles is included in a typical database, as encoded in the rs (ref Snp) number, which can contain as many as eight digits (NCBI Resources, 2013). The two alleles of a Snp are chosen from the nucleotide symbols [A,C,G,T] or equivalently as aliased by [1], [2], [3], [4]. Generally there is no particular order in the acquisition of allele pairs. Without loss of generality we adopt a convention that places the higher number first in each Snp. Since the allele content of a Snp is known from its rs number the two symbols therefore can be further aliased by 2 and 1, with the higher number going first, see Table 1 in Appendix.

**Table 1:** Nominal Snps and their transformations as described in the text.

1	Locus	…	N	N+1	N+2	N+3	N+4	…
2	Snps	…	(A,T)	(C,G)	(C,T)	(A,T)	(A,G)	…
3	Acquired Sequence	...	AT	CC	TT	TA	AG	…
4	Standard Form	…	TA	CC	TT	TA	GA	…
5	Alias	…	2 1	1 1	2 2	2 1	2 1	…

On this basis any representative sequence of a population appears as a sequence of Snps each of which contains an odd numbered and then an even numbered locus. Each allele pair then appears as: 22, 21, or 11 which gives the essential content of the Snp of a sequence. Henceforth this description, which is general and free of bias, will be referred to as the standard organization. The standard organization divides a SNP into the two allele compartments that will be referred to as odd, 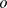, and even, 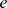. The informational content of the two compartments always exceeds the Snps form, see Appendix.

### High Value Loci

**Figure 1:**
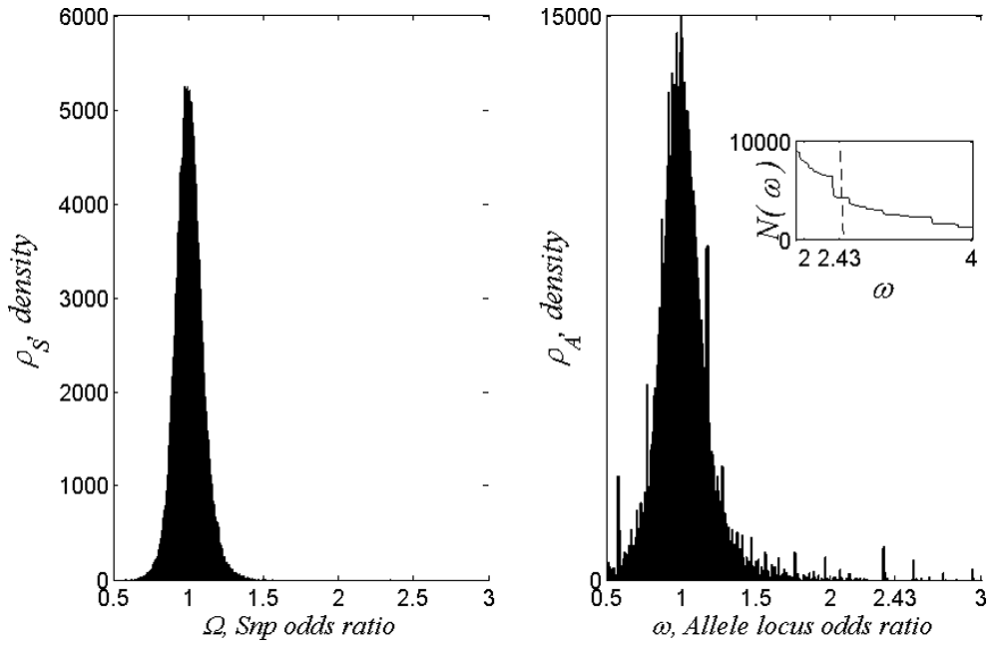
Histograms of odds-ratios for the Fusion database. At the left the customary Snps odds-ratio, 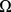, is shown, while at the right individual base pair odds-ratios, 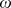, are shown. Note the ordinate range at the right is twice that at the left.

Computing challenges and rational considerations dictate an 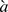 priori search for those loci that are likely to be associated with a specific disease. These will be referred to as *high value* loci. Customary treatment of GWAS data dwells on the Snp disease/control odds ratio, 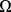, and relatively large values suggest a locus of interest. Adoption of the standard organization now permits calculation of the odds ratio for each allele, 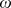. Figure 1 displays Snp and allele odds-ratios, as defined in the Appendix, for the Fusion database described in the next section. Histograms are based on 2,000 bins and viewed as densities 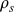 and 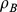 of Snps and alleles, respectively. Allele locations are far more effective locators of risk, as suggested above by their higher informational content.

### The Indicator Vector

In contrasting the disease and control populations, one must be mindful that many other variabilities are at work. For example for type 2 diabetes, T2D, specifically studied here, see next Section, the control and disease populations can be extremely diverse, since there are manifold ways of having and not having T2D, for example by possessing any number of additional diseases as well as to ethnic and other (irrelevant for us) phenotype factors. As a second step in the procedure we restrict attention to the subspace of high value loci determined from the full database. Within this sub-space a classifier is sought which is optimally correlated with the disease population, and minimally correlated with the control population, see Appendix for specific details.

**Figure 2:**
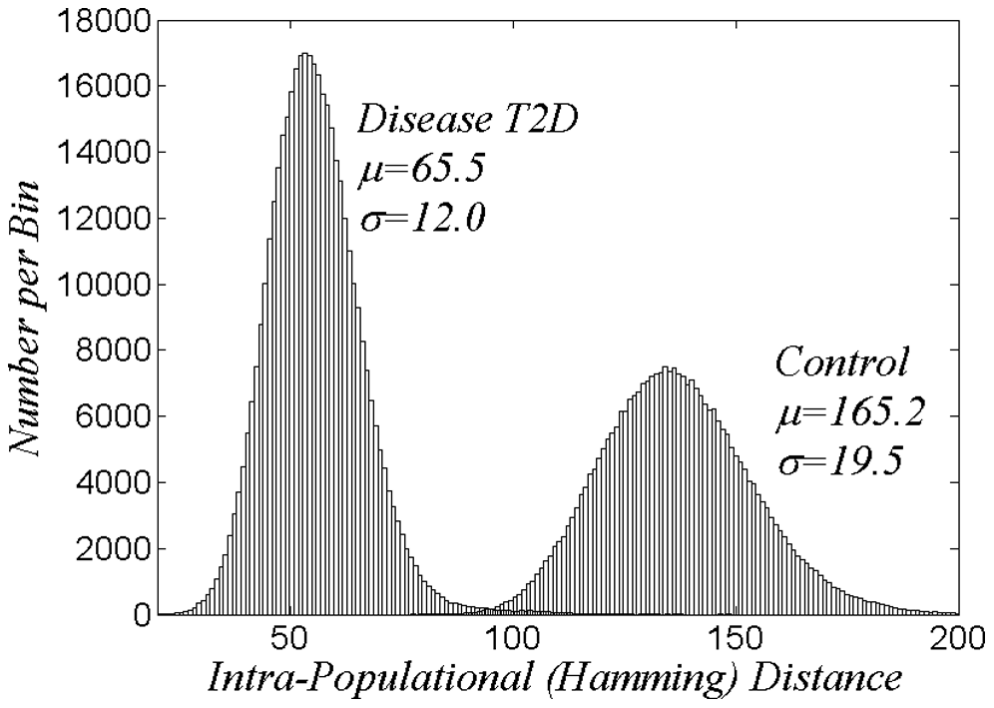
Histograms of intra-populational sequences in a sub-space of roughly 8,000 base pair loci, as obtained for odds-ratios 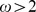 from Figure 1.

It follows from Figure 1 that there are about 8,000 loci in the subspace defined by odds ratios, 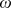, greater than 2. Figure 2 shows the histograms of all intra-population distances in this space. Distance between sequences of a population is defined as the number of letter substitutions to obtain agreement, the Hamming distance, 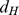. The central limit theorem suggests that both histograms are well fit by gaussians with the indicated parameters. The more widely distributed quality of the control population underlines the above remark that “there are manifold ways of not having T2D”. It is the present contention that the much more closely grouped form of the disease set, as well as the other structural differences of the two populations seen in Figure 2 provides convincing evidence that this collection of loci provides a framework that separates disease and control.

We will see that for T2D, the resulting number of loci, i.e., the dimension of the indicator is reduced but remains in the thousands. Thus the classifier is a *word* of an equal number of characters, viewed (projected) in this sub-space of allele loci.

## Application to Data: Type 2 Diabetes

Figures 1 and 2 are based on “The Finland-United States Investigation of NIDDM Genetics (FUSION) Study”, which was obtained from NIH-dbGap. This study which focuses on type 2 diabetes (T2D) has been well described in the literature [17]–[21].

This database contains 919 T2D cases and 787 normal glucose tolerant (NGT) controls. Each genomic record contained 315,693 common Snps, or 631,386 allele pairs. Although the mean level of missing data was low, .014%, individual loci had as many as 10% missing symbols. Rational procedures exist for dealing with missing data but this was not deemed to be a priority. Instead all loci which in totality had more than 2 missing symbols over all sequences were dropped. This left 272,423 Snps or 544,846 pairs. The few remaining missing symbols were then replaced by the appropriate modal symbol at the allele locus.

### Analysis Criteria

For Figure 2 the criterion for choosing high value loci was taken to be that the odds ratio, 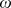, be larger than 2. This produced a set of 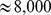 loci. Clearly Figure 2 shows a structural case/control difference. To further specify this we denote by 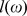 the loci set such that 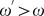 and by 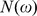 the number of these loci, shown in the inset of Figure 1. Further define 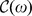 to be mode word (or mode classifier) gotten by choosing the mode symbol at each locus of 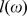. The 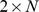 matrix
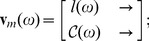
(1)will be referred to as the modal indicator (vector).

Any sequence, 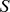, case or control, when projected on to the set 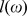, denoted by 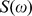, has agreement with the disease classifier given by
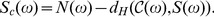
(2)


This will be referred to as the score, and clearly 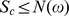.

**Figure 3:**
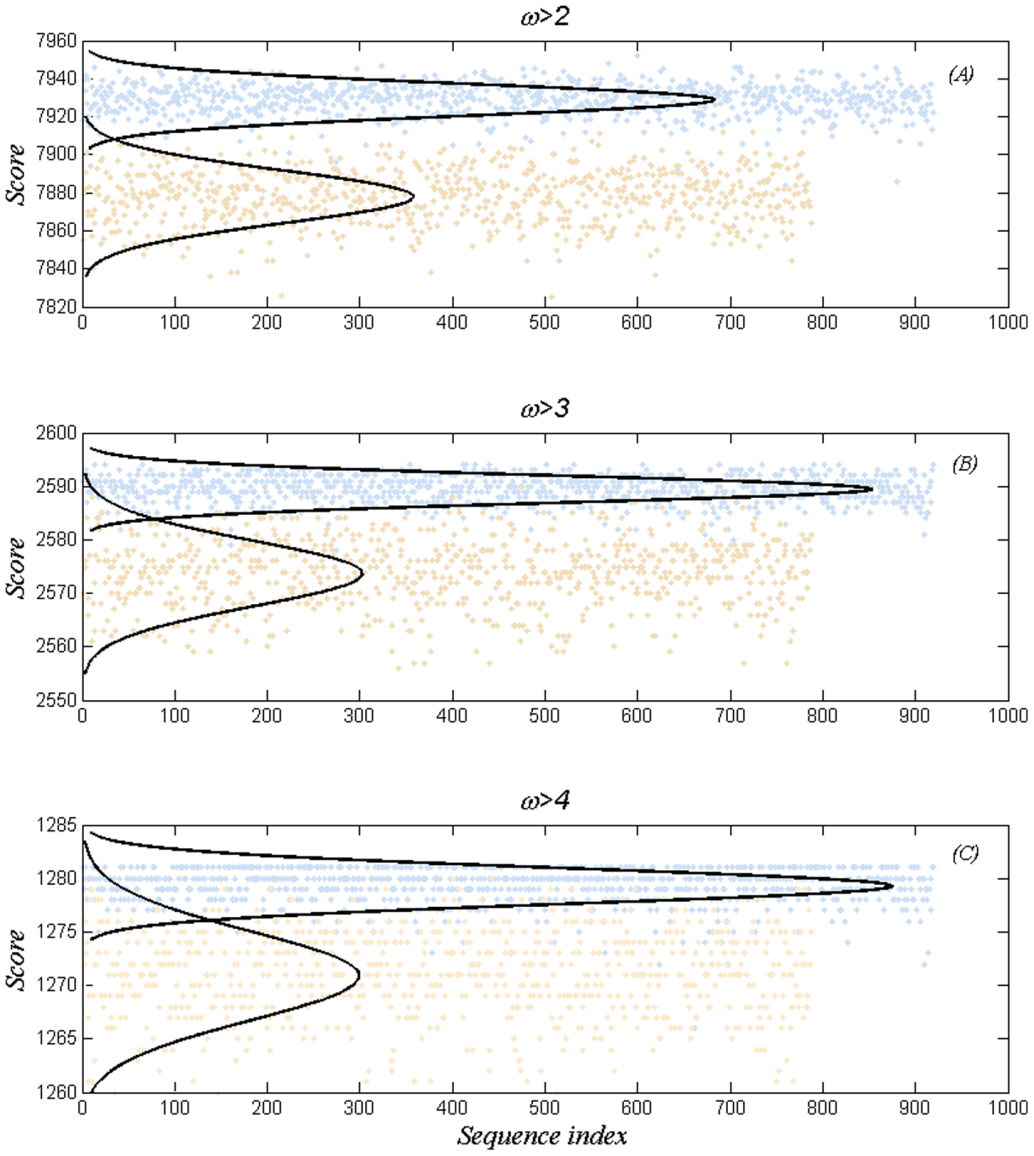
Agreement scores and gaussian fits at 3 odds ratios: (**A**) 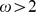, 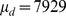, 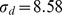, 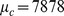, 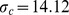 (**B**) 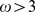, 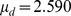, 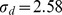, 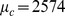, 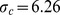 (**C**) 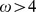, 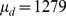, 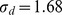, 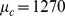, 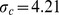. Light blue dots represent the 919 case sequences, light pink dots the 787 control sequences.

Figure 3(A) shows the scores for all 1706 sequences of T2D for 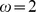; 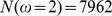, the highest possible score and as indicated the disease and control scores have mean and standard deviation 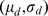 and 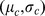 as given in the Figure legend. Since scores are sums of random variables, the central limit theorem might be regarded as applicable and the gaussian fit is also plotted on the ordinate scale. Two other cases, 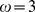 and 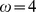, are shown in Figures 3(B) and 3(C). Across this range 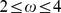 there is an accurate scaling of parameters given by
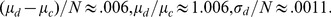
(3)but not for 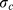.

An ROC analysis, given in the Appendix, shows that total error, false positives plus false negatives, for 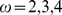 is given by 2, 18, and 66, respectively each being a relatively small fraction of the scores. The error is based on the gaussian fits; the actual data show a slightly larger error, which is due to the usual poorer fit at the tail of a distribution.

This results in nested ranges of loci which are based on the odds ratio and lead to different degrees of success in distinguishing case/control. Calculation shows that when 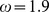 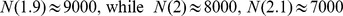 and thus there is high sensitivity in the neighborhood of 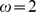, see inset of Figure 1. A detailed investigation of Figure 1 shows that 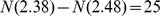 so that the interval (2.38, 2.48) is a relatively insensitive range, a sweet spot. We therefore focus on the results obtained when the odds ratio has the threshold 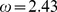, and this will be our reference case. As shown in the inset of Figure 1, 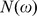, is virtually flat in this interval.

A simple argument shows that 1906 loci can precisely fit the Fusion disease/control outcomes, which raises the issue of overfitting. Figure 3(C) refutes this and larger values of 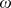 further reduce the estimate of needed loci. A later discussion will imply that two thirds of the above loci are irrelevant for T2D prediction.

### Indicator Vector

The modal symbol which appears in the mode indicator, (1) does not reflect the degree of probability of the symbol, only that it is greater than 1/2. This is improved on by the indicator vector. To obtain this we embed allele space into a Euclidean space, which has the advantage of having a distance which is an inner product, see *Indicator Vectors* in the Appendix. This choice of distance transforms the disease and control matrices, 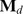 and 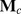, to a numerical form. Next an optimal disease classifier, 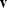, is obtained, from a reasonable criterion, see (A.13). In plain terms 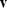 is a *word* that is highly correlated with the disease set and minimally correlated with the control set. The procedure outlined in the Appendix leads to the refinement that only loci having the most highly probable symbols are selected (see Appendix).

For the reference case with threshold 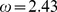, 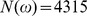 along with an estimated 6 errors. The plots for this case resemble those depicted in Figure 3. Use of he indicator vector reduces the number of loci to 4300 and an error estimate of 4. This modest reduction is largely due to the insensitivity of the odds ratio threshold in the neighborhood of 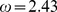. For the odds ratios shown in Figure 3, indicator reductions in the number of loci is roughly 25%.

## Validation and Prediction

Consistency of the T2D classifier is next explored by comparing it with results from data generated by randomizing the phenotypical Fusion sequences in a manner consistent with the data. The resulting classifiers computed at the same 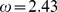 criterion level are then compared with the T2D classifier. This randomization produces three possible alterations of the 4300 distinguished loci: (1) symbol change 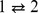; (2) high 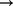 low value at a major locus; (3) low 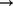 high value at a minor locus. Non-parametric statistics thus implies a 1/8 overlap of the randomized classifier with the T2D classifier, i.e., an intersection 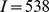, which is confirmed by the results obtained for 50 randomized trials:
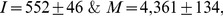
(4)where 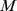 is the set size at the 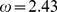 threshold. It is an underlying hypothesis of the Fusion data acquisition that the (roughly 540,000) non T2D loci should be statistically the same for disease and control. The above 3 *alterations* show that there are only T2D loci shifts. Under the randomization the overall distribution of odds ratios, 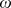, only shows changes in nearby T2D allele loci.

The results of (4) should be compared with the estimate of 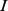 if 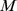 is chosen at random without replacement; in this case classifier overlaps would be given by
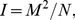
(5)where 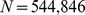.

If 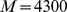, this gives 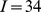, and if the symbols are also random this gives 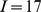. To emphasize this point 4300 loci were chosen at random and for 50 trials if the symbol is chosen as the mode we obtain 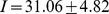; and if the symbol is chosen at random 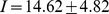, is obtained, confirming (5).

These deliberations show that the T2D classifier is certainly not only not an artifact, but also clearly emphasizes the special role of the T2D loci for type 2 diabetes.

The best evidence for the T2D indicator would be an objective test of prediction. Internal consistency was shown by splitting the Fusion data into a training set, 760 cases and 650 controls; and a test set of 159 cases and 137 controls. The indicator vector for the training set was then determined, and applied to the test set for disease/control designation. The error rate was about 1%, about the same as for the training set. Since all sequences figured in the determination of high value loci, this is irrelevant for purposes of prediction.

A successful prediction was achieved in one limiting case. At random, one disease and one control sequence was removed from the data and reserved as the ‘test set’. The classifier for the remaining data was then determined. At each locus of the classifier, the difference of the ‘correct’ classifier frequency for the disease and control populations was calculated. *For no apparent reason* if the bottom 3,000 so ranked loci replaced the original classifier a statistical advantage resulted. Over the course of 1,000 such trials a 52% correct prediction rate was obtained, yielding a 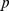-value of.03. No specific risk loci were obtained, only the certainty that such loci exist within the reduced classifier.

It is the present contention that statistical fluctuations in the data possibly allow for hidden contrasts between the case/control sets besides T2D, and that this confounds prediction. To investigate this we randomly chose sets of 60%, 70%, 80%, 90% of the Fusion populations of disease and controls. Many repeats of this showed a high level of consistency in the size of the corresponding indicator spaces. (Note this procedure always leads to a nested set of loci that at 100% is the T2D indicator that has been obtained here).

If we denote the average indicator loci size at the five values (60%, 70%,…, 100%) by 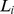 and the corresponding population sizes by 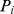 then a simple regression shows that
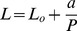
(6)with
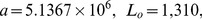
(7)fits the data to within a fraction of one percent. Thus in the limit of unbounded data the estimated number of T2D loci for the classification is 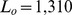. Since the degree of diversity in the Fusion data is small this is likely an over estimate, which also implies that over two thirds of the allele loci are irrelevant for prediction.

## Further Results and Discussion

For the representative case, 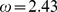, about 4300 loci were found for the classifier, however the above analysis suggests that with sufficient data this number might be reduced to about 1300. About two thirds of the 4300 are due to possible hidden contrasts, which in turn are due to data fluctuations and the limited amount of data. Unfortunately, the present analysis cannot suggest which 1300 loci are *correct*, and our further remarks can only be stated in general terms.

The literature contains suggestions that many loci figure in the genomics of complex diseases, however that thousands of genomic markers are involved might not have been anticipated. It is tempting when confronted with such large collections of loci to suggest that a network is involved, but the analysis does not have the capacity to reveal potential interactions as implied by this terminology.

**Figure 4:**
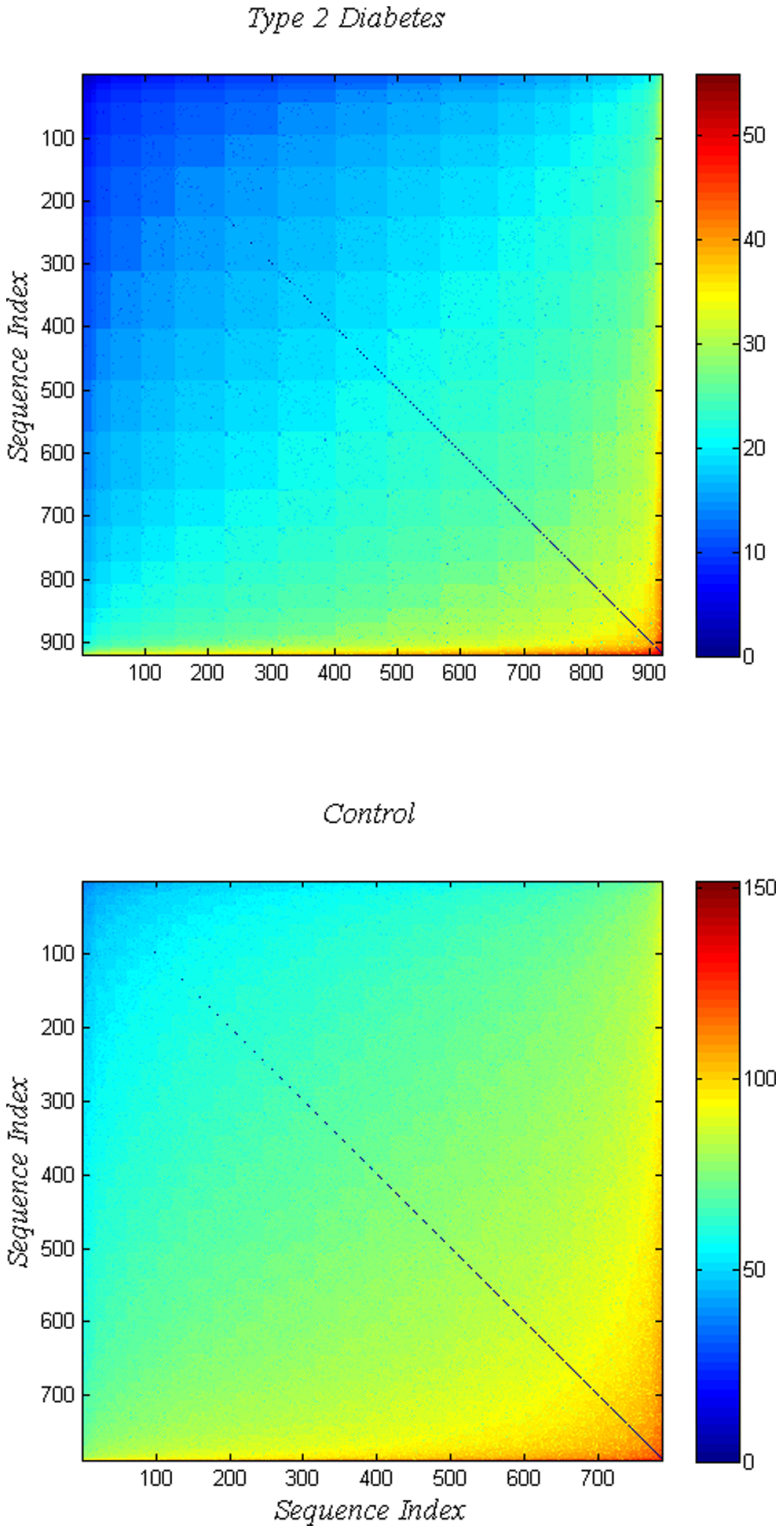
Reordering the reference case structure matrices for T2D on top, and for controls bottom. Reordering is based on ascending values of the mean Hamming distance of a sequence from all others.

To further the issue of possible patterns recall that Figure 2 was constructed from the histograms of 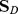 and 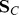 the matrices of Hamming distances of intra-disease and intra-control sequences, sometimes referred to as structure matrices. Next we adopt a re-ordering of sequences based on increasing average Hamming distance of a sequence to all others of the set; which in spirit is similar to the ordering generated by dendrograms in taxonomy. The result is shown in Figure 4 where the image of the reordered structure matrix 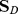 is shown above, and below that for 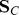. The difference in appearance of the two sets is striking; note the different color bars ranges. The upper panel of Figure 4 depicting disease shows a strong granularity and in analogy with taxonomy might indicate possible sub-types of T2D and also a lack of structure for 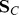.

**Figure 5:**
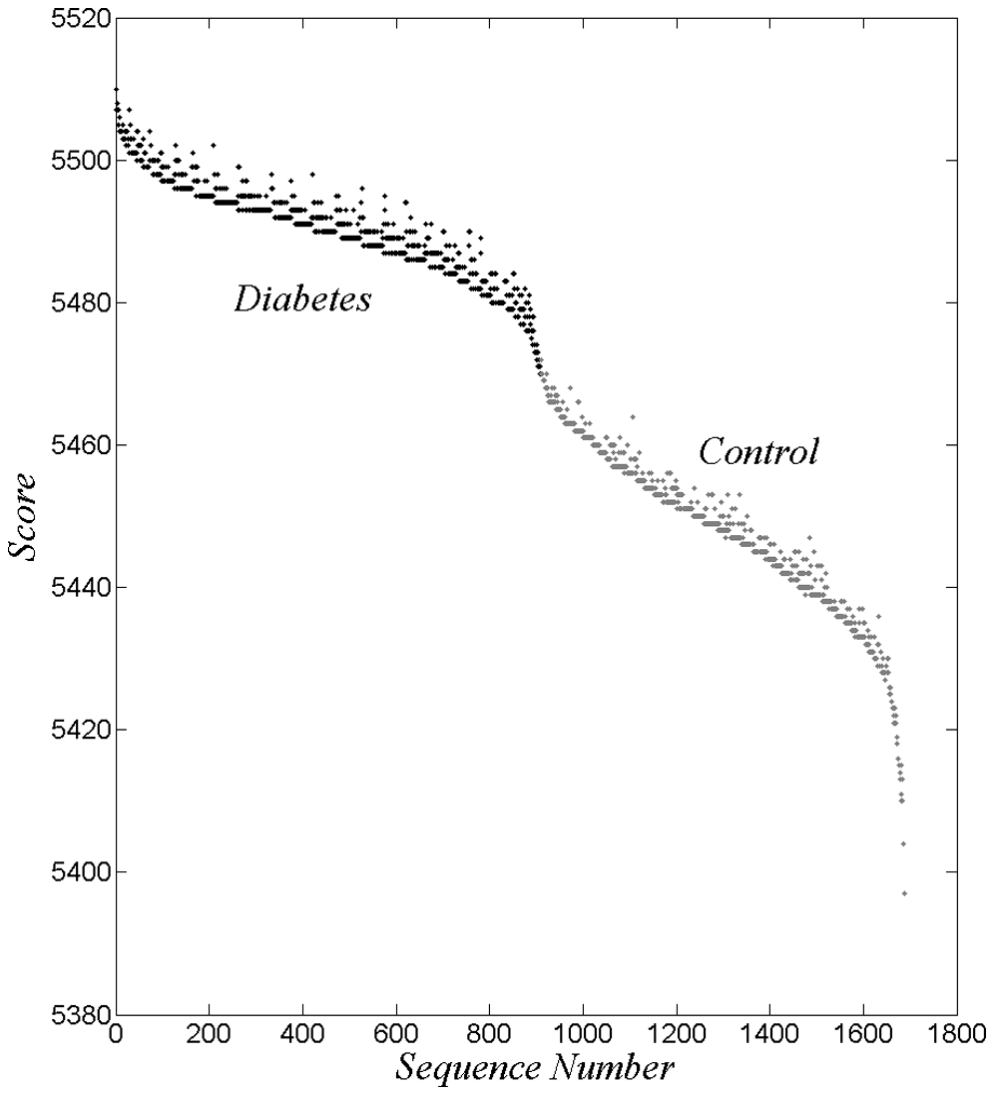
Scores reordered according to Figure 4.

Next we consider the score calculations of Figure 3 for the case of 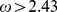, but make use of the reordering of Figure 4. This is shown in Figure 5. A small number of *false negatives* and *false positives* have been removed for display purposes. The result appears as a structured generally decreasing trace. The granularity found in Figure 4 has a counterpart in Figure 5. The steps and their vertical columns suggests nearby sets of sequences; an issue for future investigation.

The present results suggest that if the presence of Snps is due to mutations, then single mutations are irrelevant since they are shared by both the disease and control sets, an indication that attention to individual loci may be futile. The situation is consistent with the view that a threshold number of such mutations is required for disease to manifest itself; an implication of Figures 3, and 5.

As a metaphor for this situation consider the case of a neuron which gathers small incremental membrane potentials, and only when all inputs sum to a threshold does the neuron perform its function of firing an action potential. Perhaps a more relevant attempt at modeling, is to note that the distinguished collection of 4300 loci is of unknown organization, but that for a score above 4286 the collection manifests itself as diabetes, and below that as the absence of disease. In such terms the score can be compared to a morphogen, which above some threshold produces a form of tissue different than below the threshold [22], [23], with ‘function’ or ‘organization’ replacing ‘tissue’.

Recent studies of T2D data [24], [25] produced a compilation of *variants* that might be associated with T2D. In total that list contains 121 *risk loci*, but only 32 are shared with the full Fusion dataset and none of these belong to the reference set of 4300 loci. Both cited investigations made use of the observation that Snps might be acquired as segments of DNA and therefore of associated genomic material, referred to as haplotypes. This concept played no role in the present development, which regards all loci as independent. Such locational correlations might be present in the present results, and a Hapmap of the 4300 loci might prove to be revealing. Along similar lines since roughly 98% of DNA is non-coding [26] it may be of interest to determine how much of this kernel of 4300 genomic loci is coding.

The observation that a disease mechanism might not be revealed by analysis of data from genome wide associative studies [8] is not refuted by what is presented here. As already observed there have been many allusions in the literature to the modest role that individual Snps play in a disease as complex as T2D, but the notion that large numbers of loci might play a role, is probably a surprise. On the other hand since this number of loci appears to successfully distinguish disease from control it would be remarkable if the mechanism of the disease is not to be found in the set. It is also an implication of the present analysis that more case/control records would significantly shrink the classification set.

Since the distinguished kernel of genomic loci is heavily present in the *healthy* control set, a more subtle question is: ‘What is its role?’ in the control set.

## Appendix: Analytic Summary

### Standard Order

The 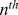 Snp of a sequence is recorded as two allele loci, say 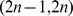 referred to as the odd and even members of the 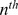 Snp pair. According to the *standard order* the higher number in the symbol alias is recorded first and is then re-aliased as 2 and the lower number is recorded second and re-aliased as 1. No information is lost since the accompanying rs number, part of the database, of a Snp furnishes chromosome location and alleles themselves. The essential Snp information is thus determined by whether 22, 21, or 11 is recorded. This is illustrated in the following table.

### Probabilities

For a population of sequences, at any Snp, one can calculate the probability 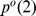 as the frequency of symbol 2 at the odd locus and similarly 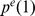 at the even locus.

It is clear from this formulation that the probability of the 22 pair, 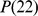 is
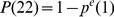
(A.1)and similarly for
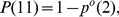
(A.2)and finally



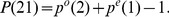
(A.3)If 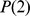 denotes the probability of symbol 2 for a Snp then
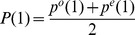
(A.4)and



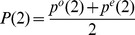
(A.5)


### Information

For the Snps case the information (entropy), from (A.5) is
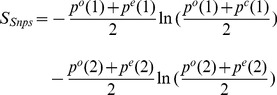
(A.6)and the allele version has two compartments


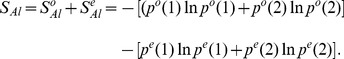
(A.7)

### Odds Ratios

For a disease and counterpart control population the allele odds ratios are given by
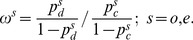
(A.8)where the subscripts 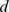 and 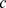 indicate the disease and control set, respectively.

The Snp odds ratio is
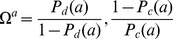
(A.9)where 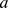 is the major allele, and in which (A.4) and (A.5) can be substituted.

Table 2 shows a string of three Snps of risk loci. The 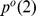 and 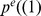 probabilities, for disease and control sets are shown on the first two lines. The last two lines show odds ratios based on allele loci and Snp loci, respectively. See Figure 1.

**Table 2:** Odds-ratios, 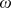 and 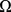, at three Snp locations.

	Snp (1)		Snp (2)		Snp (3)	
	Odd	Even	Odd	Even	Odd	Even
Disease	.2302	.9934	.2329	.9947	.9987	.1105
Control	.2415	.9754	.2354	.9785	.9923	.1000
OR *ω*	.94	**3.80**	.9862	**4.13**	**5.96**	1.118
OR Ω	1.12		1.090		.9608	

If the probabilities of risk loci lie close to unity for both disease and controls so we can write 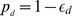 and 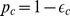 then (A.8) shows 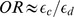, which accounts for the bold face values of line 3 of the table. On the other hand from (A.4) and (A.5) 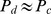 and leads to 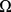 values near unity.

### Indicator Vectors

To pass from a symbolic sequence to a numerical vector we set
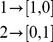
(A.10)which is a reduced form of the more general case [15], [16]. Thus



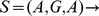


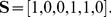
(A.11)


The transformation (A.10) of a sequence of 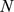 alleles becomes a vector in a Euclidean space of dimension 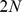. The Euclidean distances, 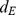, between sequences of the same length is related their Hamming distances 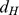 by the relation
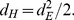
(A.12)


The vectorized matrix of disease and control sequences will be denoted by 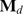 and 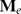 respectively, i.e., the rows of each are the vectorized genomic sequences of the corresponding data. To generate the indicator vector, 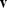, of the disease class we seek the maximum of the criterion functional
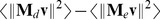
(A.13)under the condition that
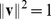
(A.14)and where 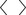 indicates the average over rows. This leads to
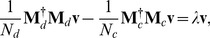
(A.15)and simple arguments show there must be at least one positive 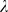.

The dimensionality of the problem can be substantially reduced by recognizing that 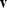 must be an admixture of the rows of 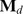 and 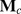, thus
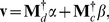
(A.16)known as the method of snapshots [27]. From (A.16) it then follows that



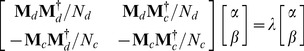
(A.17)Solution of (A.17) leads to a set of loci and the distinguished genomic symbol. As mentioned in the main text, the combination is the indicator vector and the ordered symbols, or word, is the classifier in the corresponding locus space.

### ROC Analysis

The proper odds ratio threshold might sensibly be formulated in terms of true and false positives and negatives as customarily treated by *receiver operation characteristic*, ROC, curves. For simplicity it will be assumed that the distribution of scores 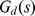 and 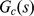, being sums of large numbers of loci, can by the central limit theorem be fit by normal distributions,
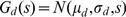
(A.18)and
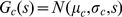
(A.19)of mean 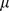 and standard deviation 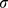.

For 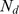 and 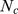 the number of disease and control sequences, and a discrimination value 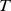


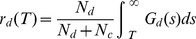
(A.20)is the fraction of true positives. The plot of this versus false positives, for the range of 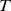 furnishes a ROC curve. The error fraction is
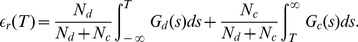
(A.21)the minimum 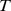, is easily calculated and occurs at the point of the ROC curve of slope −1. For any odds-ratio threshold this is considered the ideal value.

### Pseudo-probabilities

An issue is the fact that choosing the modal symbol at a locus only requires a probability 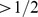. All probabilities as defined above must satisfy
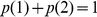
(A.22)whether defined for alleles or Snps. Under the vectorization (A.10) and the optimization (A.10) it can be shown that (A.22) is preserved. However, in the space of all probabilities, probabilities may leave the first orthant. In simple terms under (A.13) individual probabilities can be greater than unity and so also can be negative. This circumstance has a history in theoretical physics [28]–[30] and has been given a sound mathematical basis [31], and these are sometimes termed pseudo-probabilities. A part of the optimization is to only retain those loci which are overprobable, by choice the level is 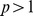, which is not critical.
